# A Proof-of-Concept Study of Transcutaneous Magnetic Spinal Cord Stimulation for Neurogenic Bladder

**DOI:** 10.1038/s41598-018-30232-z

**Published:** 2018-08-22

**Authors:** Tianyi Niu, Carol J. Bennett, Tina L. Keller, J. C. Leiter, Daniel C. Lu

**Affiliations:** 1 Department of Neurosurgery, David Geffen School of Medicine, University of California, Los Angeles, Los Angeles, California 90095 USA; 2 Department of Orthopedic Surgery, David Geffen School of Medicine, University of California, Los Angeles, Los Angeles, California 90095 USA; 3 Neuromotor Recovery and Rehabilitation Center, David Geffen School of Medicine, University of California, Los Angeles, Los Angeles, California 90095 USA; 4 Brain Research Institute, University of California, Los Angeles, Los Angeles, California 90095 USA; 5 Department of Urology, David Geffen School of Medicine, University of California, Los Angeles, CA 90095 USA; 6 Department of Surgery, Division of Urology, Greater Los Angeles VA Healthcare System, Los Angeles, CA 90073 USA; 7 Department of Molecular and Systems Biology, Geisel School of Medicine at Dartmouth, Lebanon, NH 03756 USA

## Abstract

Patients with chronic spinal cord injury (SCI) cannot urinate at will and must empty the bladder by self-catheterization. We tested the hypothesis that non-invasive, transcutaneous magnetic spinal cord stimulation (TMSCS) would improve bladder function in individuals with SCI. Five individuals with American Spinal Injury Association Impairment Scale A/B, chronic SCI and detrusor sphincter dyssynergia enrolled in this prospective, interventional study. After a two-week assessment to determine effective stimulation characteristics, each patient received sixteen weekly TMSCS treatments and then received “sham” weekly stimulation for six weeks while bladder function was monitored. Bladder function improved in all five subjects, but only during and after repeated weekly sessions of 1 Hz TMSCS. All subjects achieved volitional urination. The volume of urine produced voluntarily increased from 0 cc/day to 1120 cc/day (p = 0.03); self-catheterization frequency decreased from 6.6/day to 2.4/day (p = 0.04); the capacity of the bladder increased from 244 ml to 404 ml (p = 0.02); and the average quality of life ranking increased significantly (p = 0.007). Volitional bladder function was re-enabled in five individuals with SCI following intermittent, non-invasive TMSCS. We conclude that neuromodulation of spinal micturition circuitry by TMSCS may be used to ameliorate bladder function.

## Introduction

Spinal cord injury (SCI) leads to long-term disabilities with significant social and economic consequences. After SCI, bladder dysfunction is common and improved bladder function consistently ranks as the top quality of life priority in individuals with SCI^1,2^. Patients with a neurogenic bladder following SCI often catheterize themselves to empty the bladder, and urinary tract infections and obstructive uropathies are common^3–5^. Direct muscle stimulation^6^, stimulation of peripheral nerves^7^, or rhizotomy^8^ to restore bladder function all have limitations. Most of these interventions fail to restore the complex, orchestrated sequence of muscle contraction and relaxation that normal, voluntary micturition requires^9^.

Recently, epidural spinal cord stimulation (SCS) was used to enhance motor function in individuals with chronic SCI^10–12^. It is our hypothesis that spinal networks have the capacity to execute a range of complicated movements requiring detailed coordination among motor pools within the spine with minimal or even no input from the brain^12^, and electrical or magnetic stimulation of the spine restores or permits coordinated activation of these spinal circuits. We hypothesize that a similar mechanism of SCS to the restoration of reaching and grasping function may be at play with respect to bladder function whereby co-contraction of agonist-antagonist muscles is abolished and voluntary motor control of micturition may be restored^13^. Thus, SCS can be used to address detrusor-sphincter dyssnergia (DSD), where there is agonist/antagonist muscle co-contraction, and disinhibit or enable volitional control of the spinal micturition circuit that coordinates detrusor constriction with sphincter relaxation.

Epidural electrical stimulation can activate micturition in rodents^14^, but epidural stimulation is invasive and costly. We were able to demonstrate recently that transcutaneous electrical stimulation of the spine can activate descending motor pathways non-invasively in paraplegic individuals, but such stimulation can be painful, and the spread of electrical current may activate other susceptible structures with adverse or painful consequences^15^.

Magnetic stimulation can also be used to modulate neural circuits, and with figure-eight coils, the energy can be targeted to some extent. Moreover, transcutaneous magnetic stimulation is non-invasive and painless. Transcranial magnetic stimulation (TMS) has been used to modulate neuronal function in a variety of settings from migraine treatment^16^ to depression^17^ to restoration of motor function after ischemic stroke^18^. In the current study, we used transcutaneous magnetic spinal cord stimulation (TMSCS) to stimulate the lumbar spine to try to improve bladder function in five patients with SCI who were unable to urinate voluntarily. We hypothesized that neuromodulation of the spine using TMSCS would allow these patients to achieve voluntary micturition and reduce or eliminate the need for bladder self-catheterization.

## Results

**Figure 1:**
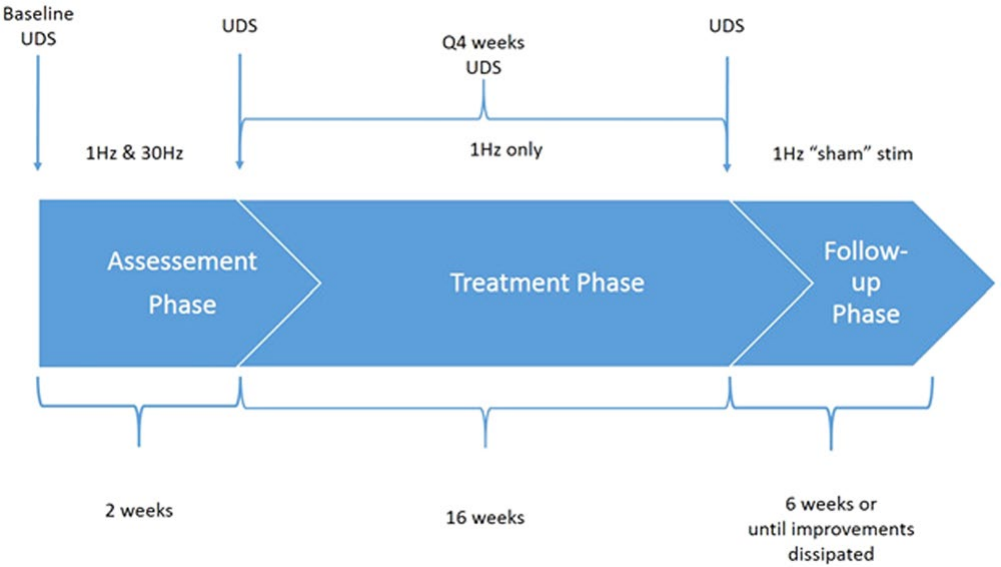
Overview of the study. There were three phases of the study: assessment, treatment and follow-up. The time frame for each is shown in the flow chart. During the assessment phase, each subject received stimulation with both 1 Hz and 30 Hz, each stimulation frequency delivered for one week, and underwent urodynamic testing (UDS) with video recording at the end of the assessment phase to determine the optimal frequency based on the changes in urethral and detrusor pressures during micturition attempts with either stimulating frequency. The 1 Hz stimulation frequency reduced urethral pressure and increased detrusor pressure in all subjects more effectively than 30 Hz stimulation. Therefore, each subject received 1 Hz stimulation during the treatment phase and received weekly stimulation treatment for 16 weeks. During the follow-up phase, the subject received “sham” stimulation at <5% intensity in order to blind each subject to the change in stimulation treatment. The follow-up phase lasted 6 weeks or until each subject’s urological improvements completely dissipated.

**Figure 2:**
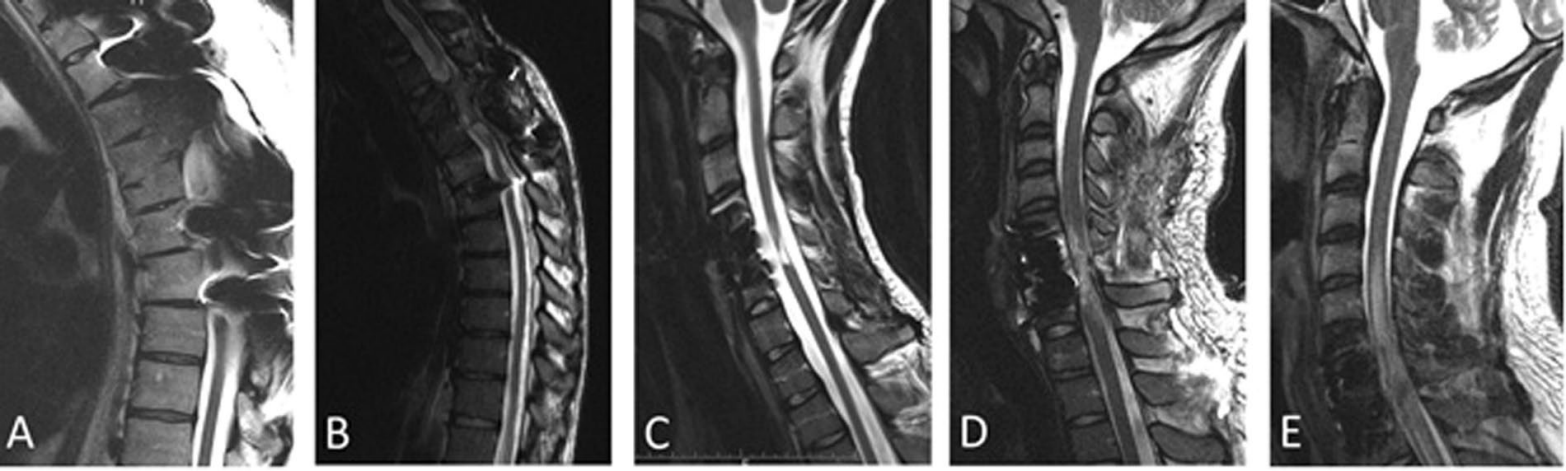
T2-weighted MRI imaging showing the degree of SCI in all five subjects enrolled in the study. The MRIs were obtained to ensure there was no spinal cord transection and to assess the anatomical level of injury (cervical/thoracic/lumbar). Authors (TN and DCL) reviewed all the MRIs prior to enrolling each subject in the study. A synopsis of the formal neuroradiology report was reviewed and included here for reference. (**A**) Prominent metallic artifact from fusion hardware in the superior to mid thoracic spine significantly obscures evaluation at these levels. The small segment in which the cord can be visualized at T4-T5 demonstrates prominent cord myelomalacia. Stable compression deformity of T5 without retropulsion. Scattered discogenic changes are seen in the thoracic spine from T8 through T12 without significant foraminal or canal stenosis. The cord is unremarkable at these levels. (**B**) Metallic artifact from instrumentation hardware in upper thoracic spine makes the evaluation of the spinal cord difficult at high thoracic spine levels. On axial images, significant myelomalacia is noted at T3-4 level. Below T5, the spinal cord appears to have normal caliber. No significant canal or foraminal stenosis. (**C**) Severe spinal cord myelomalacia at C5-C6. No evidence of spinal cord edema. Grossly stable anterior and posterior fusion from C4 to C6. Left vertebral artery occlusion, possibly related to chronic traumatic dissection. (**D**) Status post anterior fusion from C5 to C7 and posterior fusion. Metallic distortion artefact is noted through the fused C5 to C7 levels and significant myelomalacia or cord edema is noted at these levels. Visualized upper thoracic spinal cord appears to be in normal caliber with no compression. (**E**) Status post ACDF from C6 to T1 for repair of C7 burst fracture. Spinal cord edema and swelling spans from C4-T1.

Subjects underwent 3 study phases (Fig. 1). Demographic information and indices of bladder function for all five subjects are shown in Table 1. The magnetic resonance images (MRI) indicating the level and extent of SCI for each subject are shown in Fig. 2. The average duration of SCI was 8.8 ± 7.5 years. None of the subjects had been able to void voluntarily since the time of injury as shown in at least three prior urodynamic studies in each subject.

**Table 1:** Demographic information, the origin and nature of the SCI and urinary indices.

#	Sex	Age	Injury level	ASIA Grade	Disease duration (years)	Mechanism of Injury	Length of stimulation until volitional micturition (weeks)	Decay of the effect duration (weeks)	CIC/day Pre	CIC/day Post	Volitional Void (Y/N)	Stream Velocity (ml/s)	Bladder Capacity Pre (ml)	Bladder Capacity Post (ml)	Daily Voiding Volume Post (ml)
A	M	42	T4	A	13	MVA	4	4	9	0	Y	10	141	431	2000
B	M	43	T4	A	5	Wrestling	6	3	6	3	Y	10	238	462	700
C	M	22	C5	B	8	Football	5	3	6	3	Y	10	270	351	800
D	M	25	C6	B	8	MVA	5	4	6	1	Y	8	215	325	1800
E	M	23	C7	A	8	MVA	8	2	6	5	Y	8	354	452	300
Avg	—	—	—	—	—	—	5.6	3.2	6.6	2.4	—	9.3	244	404	1120
SD	—	—	—	—	—	—	1.5	0.8	1.3	1.9	—	1.1	78	62	740

AIS = American Spinal Injury Association Impairment Scale. MVA = motor vehicle accident. CIC = clean intermittent catheterization.

### Determining the optimal frequency: spinal function

**Figure 3:**
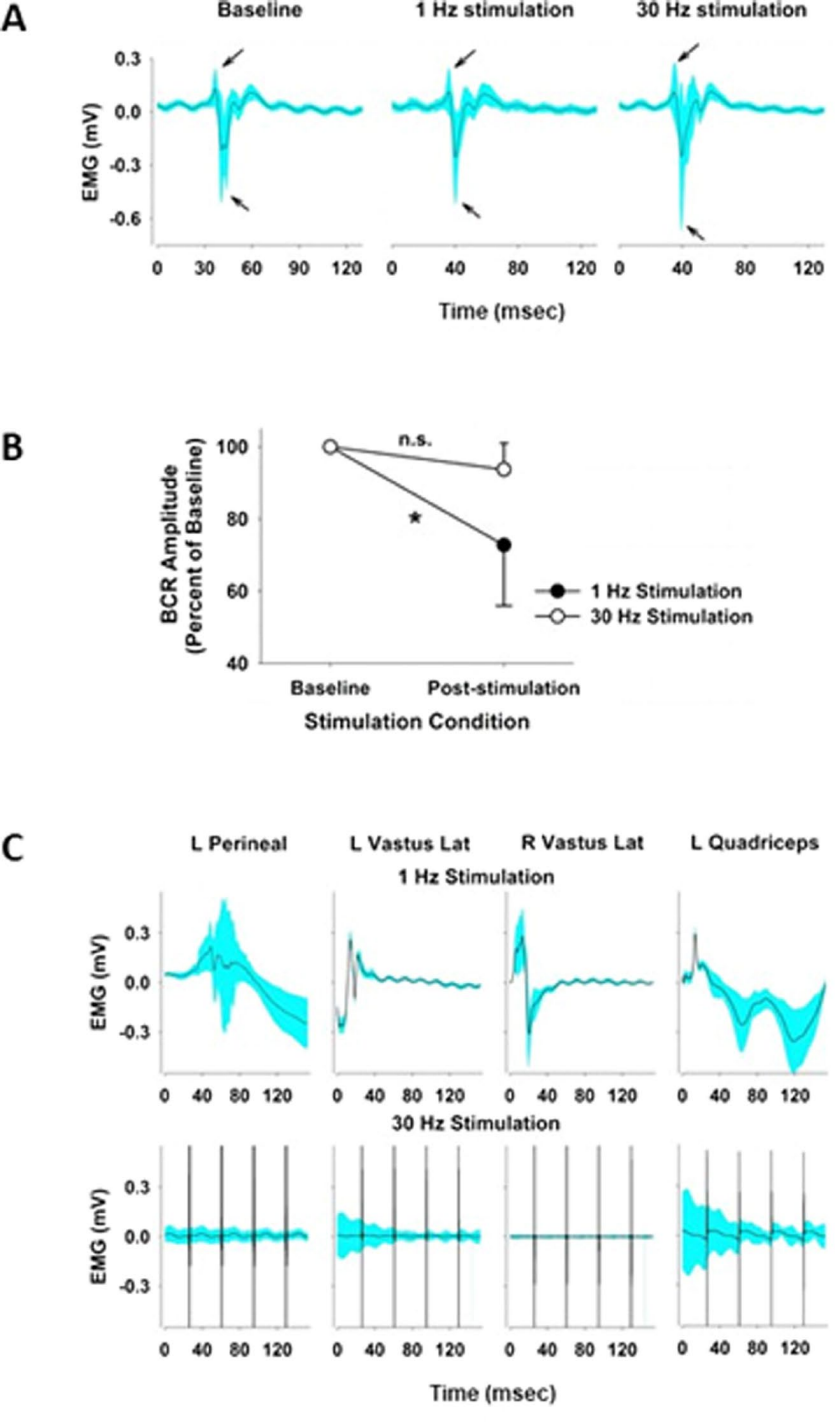
An example of the BCR amplitude (**A**), which is measured from the perineal muscle EMG activity, obtained from subject C at baseline and during low frequency (1 Hz) and high frequency (30 Hz) TCSMS of the lumbar spine at the end of the assessment phase of the study. The BCR was elicited serially >100 times, and the mean (solid black line) ± 2 times the SD (cyan shading) are shown for each stimulation condition. The average and standard deviation of BCR responses to 1 and 30 Hz TCSMS (**B**), expressed as a percent of the baseline value in each subject, are shown to illustrate that the BCR amplitude was significantly reduced during 1 Hz TCSMS compared to 30 Hz TCSMS. Student’s t-test: *p < 0.0001, n.s. = non-significant, N = 100 BCR cycles. BCR = bulbocavernosus reflex. Examples of evoked EMG activity from a single subject in selected muscles are shown in C. Lumbar TCSMS at 1 Hz elicited significant EMG activity, but 30 Hz TCSMS did not alter EMG activity. Ensemble averages of EMG activity (solid black line) ± 2 times the SD (cyan shading) were derived from greater than 100 cycles of stimulation. The stimulus artifacts are shown in the 30 Hz stimulation sequences since stimulation occurred multiple times within the recording window (large black spikes). The left (L) perineum, left vastus lateralis, right (R) vastus lateralis and left quadriceps femoris muscles were recorded. The arrows in panel A represent the peak and the nadir of the BCR.

The bulbocavernosus reflex (BCR) is disinhibited and pathologically hyperactive after SCI (Fig. 3). The BCR amplitude was significantly reduced during 1 Hz TMSCS in all five subjects (p < 0.001). In contrast, high frequency stimulation either increased the BCR amplitude or had no significant effect. The average BCR latency was 35.2 ± 5.3 ms during both 1 Hz and 30 Hz TMSCS, which is similar to the latency of the BCR in normal individuals^19^.

During 1 Hz TMSCS, spinal cord evoked potentials could be elicited in selected lower extremity muscle groups (perineal, vastus lateralis and quadriceps femoris); whereas we were unable to detect any spinal evoked potentials at 30 Hz stimulation (Fig. 3).

### Determining the optimal frequency: bladder function

**Figure 4:**
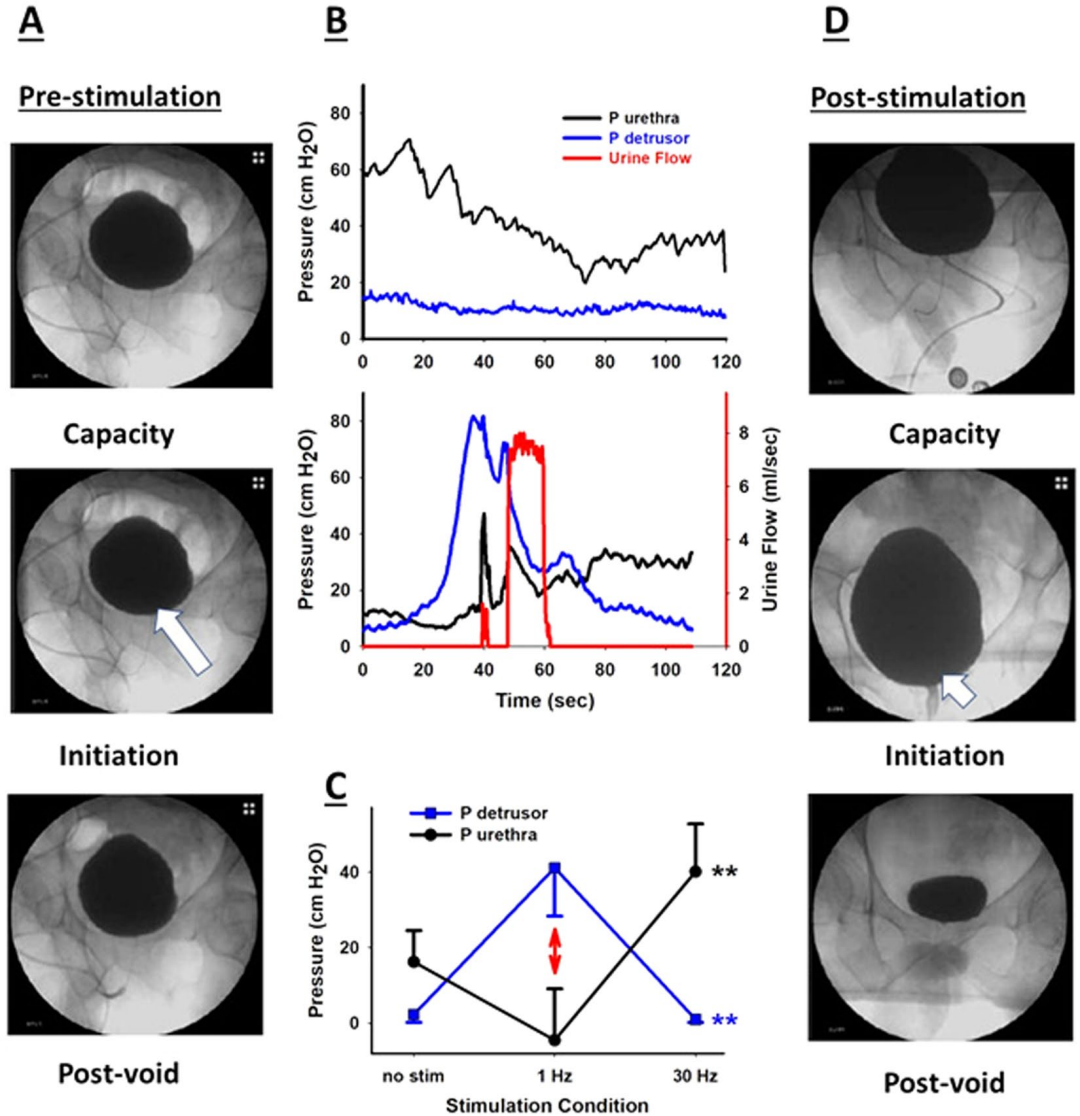
Examples of video urodynamics are shown from patient A (panels A – before the 16-week TMSCS treatment and D – after the 16 week TMSCS treatment). The first video images in each sequence show the pre-voiding bladder capacity, which increased after TMSCS. The second images show the initiation of volitional voiding and opening the bladder neck (white arrows), and the final images show the post-void residuals. In panel B, examples of urine flow (red line); urethral pressure (black line) and detrusor pressure (blue line) are shown before (upper graph) and after the 16-week TCSMS treatment (lower graph). Note that detrusor pressure remained below urethral pressure before TMSCS, and no urine flow was generated; whereas detrusor pressure exceeded urethral pressure and urine flow was generated after 16 weeks of TMSCS. The average urethral and detrusor pressures ± SD obtained during efforts to void at the end of the assessment phase are shown in panel C during baseline and 1 and 30 Hz TMSCS. The detrusor pressure rose significantly and the urethral pressure fell significantly only during 1 Hz TMSCS compared to the non-stimulated condition and the 30 Hz condition (**p < 0.0001), but the baseline, unstimulated state and the 30 Hz condition did not differ from each other based on an ANOVA and specific comparisons using Tukey’s HSD.

During the assessment phase, the urethral (P urethra) and detrusor pressures (P detrusor) obtained during urodynamic testing during attempted volitional micturition were significantly different during high and low frequency TMSCS (Fig. 4). The urethral and detrusor pressures are shown in Tables 2 and 3, respectively. The average urethral pressure was significantly lower than the baseline, unstimulated value during 1 Hz stimulation (p < 0.05) and the average urethral pressure was greater than the unstimulated, baseline value during 30 Hz stimulation (though this was not statistically significant P < 0.10). On the other hand, the average detrusor pressure was significantly elevated during 1 Hz stimulation compared to both the baseline, unstimulated condition and 30 Hz stimulation (P < 0.01 for both comparisons), and the detrusor pressure was not different from baseline conditions during 30 Hz stimulation (p = 0.5). Thus, stimulation at low frequency allowed each subject to elevate the bladder pressure and reduce the urethral pressure (conditions conducive to urine flow), and 30 Hz stimulation had the opposite effect: urethral pressure increased significantly, but detrusor pressure was not modified by 30 Hz stimulation (Tables 2 and 3). Not surprisingly, increasing detrusor contraction and bladder pressure while simultaneously decreasing urethral pressure allowed voluntary micturition (Fig. 4).

**Table 2:** At the end of the assessment phase, changes in urethral pressure in five subjects during micturition attempts compared to the pre-attempt baseline.

**Δ P ure (mmHg)**	No stimulation (NoS)	Low frequency (1 Hz)	High frequency (30 Hz)
Subject A	28.4 ± 5.0	−25.3 ± 2.7	36.2 ± 11.1
Subject B	2.5 ± 4.8	0.4 ± 5.2	50.3 ± 17.0
Subject C	19.8 ± 3.4	−8.3 ± 6.3	20.0 ± 10.0
Subject D	18.9 ± 1.5	5.6 ± 7.3	27.6 ± 4.1
Subject E	15.3 ± 5.2	−0.9 ± 9.6	46.6 ± 11.1
Average	17.0 ± 9.4	−5.7 ± 12.0 1 Hz vs NoS p < 0.05 1 Hz vs 30 Hz p < 0.001	36.1 ± 12.7 30 Hz vs NoS p < 0.10

Positive numbers indicate an increase in the pressure during the attempts while negative numbers indicate a decrease in the urethral pressure during the attempts. Notice that high frequency stimulation (30 Hz) resulted in increased/unchanged urethral pressure during attempted micturition when compared to the no-stimulation/baseline; low frequency stimulation (1 Hz), on the other hand, significant decreased urethral pressure compared to both unstimulated and 30 Hz stimulation conditions.

**Table 3:** At the end of the assessment phase, the change in detrusor pressure in five subjects during micturition attempts compared to the pre-attempt baseline.

**Δ P det (mmHg)**	No stimulation	Low frequency (1 Hz)	High frequency (30 Hz)
Subject A	19.4 ± 2.2	38.8 ± 0.9	24.5 ± 4.5
Subject B	26.1 ± 1.5	28.2 ± 3.7	0.6 ± 1.3
Subject C	1.3 ± 0.6	30.4 ± 4.3	1.3 ± 0.9
Subject D	3.0 ± 1.5	49.4 ± 19.5	1.6 ± 0.8
Subject E	−1.1 ± 0.7	58.5 ± 9.4	−0.1 ± 0.7
Average	9.7 ± 12.2	41.1 ± 12.8 1 Hz vs NoS p < 0.001 1 Hz vs 30 Hz p < 0.001	5.6 ± 10.6 30 Hz vs NoS p = 0.50

Positive numbers indicate an increase in the pressure during the attempts while negative numbers indicate a decrease in the pressure during the attempts. Notice that high frequency stimulation (30 Hz) did not change in detrusor pressure during attempted micturition when compared to the no-stimulation/baseline; low frequency stimulation (1 Hz), on the other hand, significantly increased detrusor pressure compared to the non-stimulated and 30 Hz conditions.

Based on the BCR response, the evoked EMG activity and the responses of urethral and detrusor pressures, only 1 Hz TMSCS was used for weekly TMSCS during the treatment period.

### Bladder function before, during and after TMSCS

**Figure 5:**
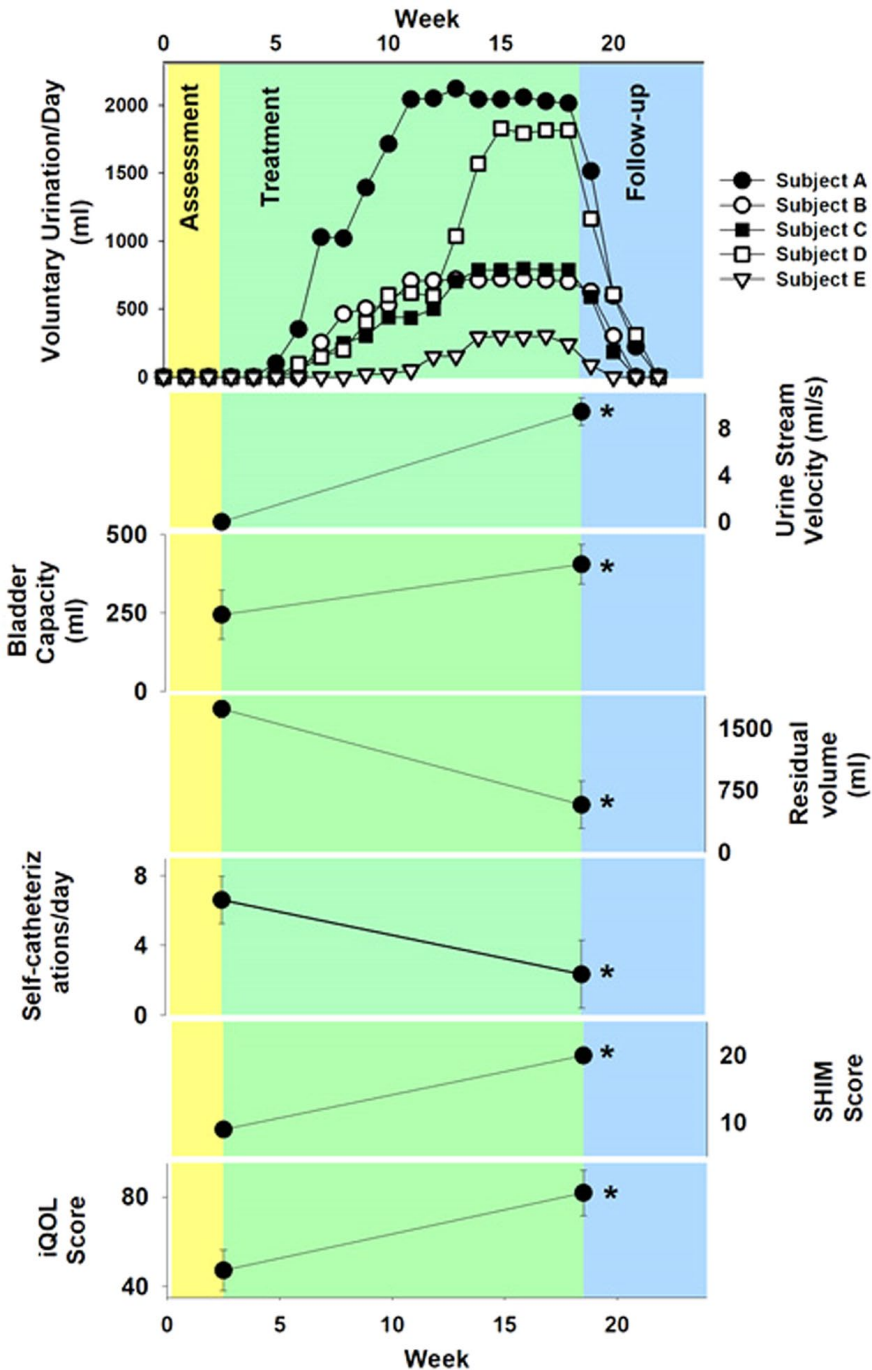
Summary of urological functions for all five subjects and average daily volitional micturition volume for all five subjects during follow-up phase; all changes were statistical significant when tested with paired t-tests (p < 0.05; see Results for details). The top panel shows the timing of recovery and loss of voluntary control of micturition and the volume of urine produced each day as a function of time. All five subjects recovered the capacity to urinate voluntarily, and about 2–3 weeks after the termination of TMSCS, the capacity to urinate voluntarily declined rapidly back to the baseline (unable to void voluntarily). The remaining panels indicate the initial value of each variable before the start of TMSCS and after 16 weeks of TMSCS. The urine stream velocity and the bladder capacity (both measured during urodynamic studies) increased significantly (p < 0.05) after 16 weeks of TMSCS. The residual volume and the number of self-catheterizations diminished significantly (p < 0.05, for both variables), and the SHIM Score and the iQOL, both quality of life measures, increased significantly (p < 0.05) after 16 weeks of TMSCS.

All five subjects achieved at least some volitional urination following 16 weeks of bladder rehabilitation with TMSCS (Fig. 5). No subject achieved volitional urination until at least 4 weekly TMSCS treatments had been given, and the capacity to urinate voluntarily was restored in all 5 subjects on average 5.6 ± 1.5 weeks after TMSCS was begun. The capacity to urinate voluntarily was maintained throughout the 16-week treatment period.

Daily self-catheterization decreased from 6.6 times per day at baseline to 2.4 times per day at the conclusion of the 16-week bladder rehabilitation (p = 0.04). Based on urodynamic studies conducted at the end of the TMSCS treatment, the average volume of urine generated voluntarily increased from 0 cc/day to 1120 cc/day (p = 0.03), and the subjects were able to generate significant urine stream velocities, which rose on average from 0 cc/sec to 9.3 cc/sec (p < 0.001). The bladder capacity increased from 244 ml to 404 ml (p = 0.02). Sexual function also improved from 9 to 20 as measured by Sexual Health Inventory for Men (SHIM) (p = 0.0003). The subjects enjoyed a much higher quality of life; the average i-QOL score rose from 47 to 82 (p = 0.007, Fig. 4). While all five subjects had improved bladder function and were able to achieve volitional micturition, their responses to TMSCS varied (the responsiveness order was A > D > B = C > E). This variation did not appear to be the result of differences in their AIS. (Table 1).

The average time that volitional micturition was maintained after the sham stimulation began was 3.2 ± 0.8 weeks. Follow-up diary entries confirmed that the ability to void voluntarily rapidly decayed in all subjects after the cessation of effective TMSCS, and no subject maintained the capacity for voluntary micturition five weeks after the last effective stimulation.

## Discussion

Voluntary micturition requires complex, orchestrated neuromuscular control of the urinary bladder by sensory, motor and autonomic systems. During voluntary micturition, sympathetic inhibition of bladder contraction is withdrawn, parasympathetic activation of the detrusor contraction emerges to increase vesicular pressure, and contraction of the urethral sphincter is inhibited to allow urine to flow out of the bladder. This control is achieved through fronto-pontine-spinal cord projections to parasympathetic ganglia in the abdomen and to sympathetic and somatic neurons in the caudal spine. In individuals with SCI, coordination among parasympathetic, sympathetic and somatic nerve activities is lost: bladder pressure is elevated, but the bladder cannot be completely emptied because contraction of the external sphincter is not inhibited. Patients with SCI must perform multiple bladder self-catheterizations each day to evacuate urine and to prevent kidney injury due to high pressure; which increase the risk and frequency of infection and traumatic injury to the urethra. Any decrease in catheterization frequency, which was achieved in all study subjects, represents a potential decrease in complications associated with catheterization.

Isolated regions of lumbosacral spinal cord contain circuits that are capable of carrying out complex motor activities^20,21^. Furthermore, spinal cord injury in most motor complete, AIS A and B SCI subjects is not anatomically complete, and many spinal circuits remain intact, especially those below the level of the spinal cord injury^22^. In both animal and human subjects with chronic paralysis from SCI, motor movements have improved after invasive, epidural, electrical stimulation^10–12^. In this study, we hypothesized that the spinal micturition circuit remains intact in subjects with SCI, and since this circuit is semiautonomous, we should be able to enhance activation of patterned muscle activities controlled by these circuits and activate or modulate them using TMSCS over the thoracolumbar spine. The mechanism of action appears to be similar to the use of stimulation to improve upper extremity function in which the threshold of motor circuit activation is diminished to enable volitional, coordinated agonist-antagonist muscle activity^13^. Voluntary bladder control was restored to some extent by TMSCS in all five individuals with chronic SCI. Four out of five subjects (80%) were able to decrease the frequency of self-catheterization by at least 50%. One subject was able to void normally without any self-catheterization while another subject only needed one catheterization each day (Table 1).

Other attempts to restore urination in SCI patients by stimulating multiple peripheral nerves, specifically the pudendal, pelvic, hypogastric and tibial nerves^23–25^, did not consistently improve bladder function. Furthermore, sacral nerve modulation requires electrode implantation, which is invasive and risky^26,27^. TMSCS differs in that it is non-invasive and painless in patients with SCI. In addition, TMSCS provides more consistent and effective bladder emptying than existing epidural stimulation of selected peripheral nerves^6–8^.

We believe that TMSCS allowed volitional activation of a coordinated pattern of parasympathetic withdrawal and sympathetic activation and somatic muscle inhibition as demonstrated in urodynamic studies. While the precise mechanism of TMSCS remains unknown, the coordinated activity of detrusor and sphincter muscles suggests that TMSCS works by activating or enhancing activation of central pattern generating circuits within the lumbosacral spinal cord and does not rely solely on activation of motor neurons or peripheral nerves. This hypothesis receives further support from the divergent responses to TMSCS at 1 Hz and 30 Hz: 1 Hz TMSCS resulted in decreased urethral pressure, increased detrusor pressure and micturition, as opposed to 30 Hz TMSCS, which increased urethral pressure, decreased detrusor pressure and enhanced urine storage within the bladder. The different stimulation frequencies elicited different bladder behaviors as if different central pattern generators (CPGs) or different aspects of a micturition CPG were activated. These divergent responses suggest that TMSCS may be applicable to a broader range of conditions such as hyperactive bladder, which may benefit from higher frequency stimulation.

We selected patients with detrusor-sphincter dyssynergia specifically because this is the group of SCI patients most recalcitrant to treatment. A regular schedule of self-catheterization prevents ureteral reflux and the development of obstructive uropathy and chronic renal failure, but frequent catheterization has risks of its own: infection, creation of false passages, urethral stricture^28,29^, a reduced quality of life, and a loss of independence. Improving quality of life is our ultimate goal using TMSCS, but this cannot be achieved if the risk of ureteral reflux and chronic renal failure increases. Therefore, any benefits of TMSCS, such as a more physiological voiding sequence with low storage pressures and increased bladder capacity and a better coordination of increased detrusor compliance and reduced external sphincter pressures that enable unobstructed voiding in a low pressure system, will be beneficial in the long term only if ureteral reflux is not increased. Video urodynamics performed at the initiation and termination of our study demonstrated no evidence of reflux. While this was a proof of concept, pilot study with patients followed for 16 weeks, additional studies are needed in an expanded cohort with extended follow-up to ensure that stable bladder and renal function are maintained when TMSCS is used to increase voluntary micturition and reduce the frequency of self-catheterization.

The BCR is a polysynaptic reflex, and BCR amplitudes in our subjects were 10 to 100 times larger at baseline than in normal individuals. Hyperactivity of the BCR may be analogous to the hyperactivity of tendon reflexes following SCI and suggests that subjects with SCI have decreased supraspinal inhibition of the BCR. During low frequency TMSCS, the amplitude of the BCR decreased, from which we infer that TMSCS induced greater inhibition of the BCR. Magnetic stimulation may achieve these effects by modulation of spinal interneurons via dorsal root ganglion or dorsal column stimulation, which is a putative mechanism of action for epidural spinal cord stimulation^30^, or TMSCS may modulate responses within the sympathetic chain and sacral parasympathetic centers and facilitate the process of micturition.

Improvements in urinary function were not instantaneous; progressive improvement became apparent over the course of the study. Initially, simultaneous measurements of urethral and bladder pressures during volitional urination attempts revealed little (if any) sustained bladder contraction and persistently elevated urethral pressures, but after completion of at least 4 weeks of effective TMSCS, subjects became better able to generate sustained bladder contractions although detrusor-sphincter dyssynergia persisted (increased bladder pressures, but also increased urethral pressures, which prevented bladder emptying). At the end of the 16-week rehabilitation period, subjects were able to produce voluntary, coordinated bladder contractions with high detrusor pressures and reduced urethral pressures. Since bladder pressure exceed urethral pressure, urine flow velocity was increased and significantly higher urine volumes were achieved (Fig. 2**)**.

Our subjects were able to urinate voluntarily in between treatment sessions when magnetic stimulation was not present. We believe that TMSCS persistently raised the activation state (or reduced inhibition) of the micturition circuit so that residual neural pathways between the supraspinal micturition centers and lumbosacral micturition central pattern generators were re-invigorated, which is consistent with previous findings using epidural stimulation to enhance recovery of motor function^12^. Restoration of voluntary micturition required repetitive TMSCS over at least 4 weeks. The benefits of epidural electric stimulations on motor function also required 3–5 sessions/weeks before improvements in motor functions were seen^12^. Once supraspinal to spinal communication had been restored or re-enabled by TMSCS, it remained enabled so long as the subject received some minimal amount of TMSCS during each weekly treatment session, but the benefits of TMSCS were not permanent. All subjects lost the ability to control micturition soon after the termination of effective TMSCS (Fig. 5). The temporal dynamics of the onset and offset of benefit of TMSCS are consistent with remodeling of the spinal circuitry in which some relatively slow neuronal or circuit remodeling is required to re-establish effective synaptic or supraspinal communication^31–33^, and some aspect of TMSCS was necessary between periods of volitional bladder emptying to maintain the integrity of communication between supraspinal and lumbar micturition circuits. The once weekly treatment interval and stimulation protocol represent a surprisingly small recurrent input to maintain volitional micturition, but this schedule is feasible for patients, and TMSCS could be administered in weekly physical therapy sessions at low cost. In any event, neuronal plasticity or remodeling are well recognized in TMS studies, specifically with low frequency (1 Hz) stimulation^34,35^. These results and our study of hand function^12^ provide two examples of the capacity of neuromodulation of spinal circuits to enable volitional control of motor functions below the level of SCI.

The responses to TMSCS varied among our five subjects. While we do not have a precise explanation for this, we know that the variation was not a result of differences among the AIS (Subject A, B, E were all category A, but subject A improved much more than the other two). The reasons for the variation are likely multifactorial, but perhaps most importantly, our subjects have variable amounts of residual spinal function. The current AIS is not sensitive to the subtleties of residual spinal functions among subjects.

The main limitations of our study are its small size and the lack of proof of the actual mechanism of action. As this is a pilot study, we plan to continue to expand the study and enroll additional subjects. Further studies will focus on the molecular and cellular processes that follow magnetic stimulation to investigate the precise mechanism of action of magnetic stimulation.

## Methods

### Subject selection

We conducted a pilot, prospective, interventional study in five subjects. All aspects of the study were approved by the UCLA IRB (IRB# 14-000932) and filed with ClinicalTrials.gov (registration number: NCT02331979, date of registration: 06/01/2015). All methods were performed in accordance with the relevant guidelines and regulations as stipulated by UCLA IRB. Informed consent was obtained prior to subject participation. The inclusion criteria for the study were male age 18–75, a stable American Spinal Injury Association Impairment Scale (AIS) A/B, motor complete spinal cord injury between spinal levels C2-T8 present for greater than 1 year, and a documented history of neurogenic bladder requiring intermittent catheterization. Each subject was required to have at least three prior urodynamic studies to confirm the diagnosis of neurogenic bladder with detrusor sphincter dyssynergia (DSD), which was diagnosed with urodynamic study in which a rise in detrusor pressure and concomitant needle EMG activity and rise in urethral pressure were demonstrated (see Tables 2 and 3). Patients with a history of autonomic dysreflexia were excluded from the study. Any patient who was ventilator dependent, abusing drugs, had musculoskeletal dysfunction (i.e., unstable fractures), cardiopulmonary diseases, active infections or ongoing depression requiring treatment, or had previous exposure to and use of spinal cord stimulation was excluded from the study. Patients with a history of bladder botox injection or bladder/sphincter surgeries were excluded. Five subjects were recruited and completed the study. There was no subject attrition.

### Intervention

Each study subject underwent baseline urodynamic testing (UDS) at the beginning of the study to confirm the diagnosis of a neurogenic bladder with DSD and establish baseline bladder functions. The study was divided into three phases: an Assessment phase (2 weeks), a Treatment phase (16 weeks) and a Follow-up phase (6 weeks). During the Assessment phase, each subject underwent once/week transcutaneous magnetic spinal cord stimulation (TMSCS) at both 1 Hz (low) and 30 Hz (high) frequency (40–60% intensity) over the lumbar spine (described below). 1 Hz and 30 Hz were both administered during the assessment phase because the optimal stimulation frequency in human subjects was unknown to us prior to this study. The two frequencies were administered in random order. These frequencies was chosen based on previous results in animals in which low frequency stimulation promoted and high frequency stimulation inhibited micturition in animals with SCI^13^. At the conclusion of the Assessment phase, each subject underwent another UDS to determine the better stimulation frequency (the characteristics of optimal stimulation are defined below). Once the better frequency was established (and it turned out that 1 Hz was better than 30 Hz stimulation in all five subjects), each subject entered the treatment phase of the study and received weekly transcutaneous lumbar spinal cord magnetic stimulation for a total of 16 weeks (described below). This 16-week period of TMSCS constituted bladder rehabilitation. Each subject received non-video urodynamic testing once every four weeks during the treatment phase to monitor progress and insure that bladder function was not further impaired. After the initial four-week stimulation period, each subject was asked to attempt volitional urination for 5–10 minutes prior to bladder catheterization. The subjects were instructed to keep the environment quiet, relax and focus on voiding. Specifically, they were instructed to perform no straining/Valsalva maneuver, external compression by Crede maneuver, reflex triggering by tapping, anal stretch, or pushing. Attempts were limited to 10 minutes. Each subject was given a urine/stool specimen collection pan (Medline DYND36600H, Mundelein, IL) to collect any volitional urinary output. In order to prevent potential urinary retention, the subjects were asked to self-catheterize after the volitional attempt and to record the catheterization output. The urinary output and the volume of the residual urine in the bladder (collected after attempted void by each patient’s routine bladder catheterization) were recorded in a diary after every attempt to urinate voluntarily. Each subject was also asked to record any other changes that he may have noticed in the diary throughout the study period. During the follow-up period, sham transcutaneous magnetic stimulation (sham) was employed at reduced intensity (5%), which replicated the auditory, partial sensory and mechanical cues of real stimulation. Each subject was instructed to continue to attempt to urinate voluntarily as he had during the treatment phase, and each subject continued to keep a detailed urological lifestyle diary until the end of the follow-up phase (Fig. 1).

Each subject was also given an incontinence quality of life (iQOL) questionnaire to complete prior to the start of the study and at the end of the 16-week treatment stimulation. iQOL has been validated in multiple urological quality of life studies in patients with SCI^36,37^. Sexual functions were assessed by Sexual Health Inventory for Men (SHIM) questionnaire^38^ at the beginning of the study and at the end of 16-week treatment phase.

### Blinding

The state of knowledge at the start of the study, in which we did not know the effective parameters of stimulation to effect micturition, precluded a randomized trial. Therefore, we conducted a single arm study in which each subject acted as his own control. Additionally, a sham phase was conducted at the end after stimulation because there was exposure to stimulation during initial Assessment Phase (we had no initial, baseline, non-stimulated collection period), and we were unsure of the wash-out period for this exposure due to the pilot nature of this study. However, subjects and experimenters were blinded throughout the process in the assessment, treatment and follow-up phases. Given that these spinal cord injury patients have diminished/no sensation due to their injuries, The subjects did not feel any sensations at 1 Hz or 30 Hz at the level of stimulation used during treatment as they have muted sensory capacity due to their spinal cord injury. We purposefully selected a relatively low intensity stimulus to avoid any painful sensations, and the stimulation level was below the sensory and motor threshold. The subjects did hear a “click” during each stimulation (especially at 1 Hz when the click was very predicable). This auditory cue was re-created during sham stimulation as well. Approximately from T11-L3 level. The coil dimensions are 172 × 92 × 51 mm in a figure-of-eight formation with two rings each 75 mm in diameter. The target (focality) is the center of the figure of eight, which in our case is T12-L1 area, which overlies the conus medullaris in humans. We used a research coil (with identical sham and treatment faces), which allowed blinding of both experimenter and subject; thereby double blinding the follow-up phase of the study. The staff member responsible for controlling of the stimulator and the dose of stimulation was not blinded as the stimulation parameters were manipulated during the various phases of the study; however, this person did not interact with the subject (he sat behind a curtain), and each staff member was instructed to follow the same script when administering the various tasks regardless of the particular stimulation values used. To assess integrity of blinding, we asked subjects at the conclusion of the study what each study phases consisted of; their responses were no better than chance.

### Urodynamic testing

We employed a commercially available urodynamic machine (Laborie Aquarius® XT, Laborie International, Mississauga, ON, Canada). Prior to the urodynamic testing, each subject emptied his bladder by direct catheterization. The volume of urine was recorded. The patient was then placed in a supine position and a triple lumen catheter (TLC-7M, Laborie International, Mississauga, ON, Canada) was inserted. Two needle recording electrodes (1512A-M, Laborie International, Mississauga, ON, Canada) were inserted bilaterally into the perineal muscles approximately halfway between the base of the scrotum and anus and 1 cm lateral to the midline. An EMG grounding pad was placed on the knee joint. A rectal catheter (RPC-9, Laborie International, Mississauga, ON, Canada) was inserted to record abdominal pressure. The subject was next placed in a left decubitus position. A condom catheter was used to collect any urine output, which was directed through a funnel into a graduated cylinder (DIS173, Laborie International, Mississauga, ON, Canada) on a scale (UROCAP IV, Laborie International, Mississauga, ON, Canada) to record the volume of urine produced and the stream velocity.

### Transcutaneous magnetic stimulation

A MagVenture Magnetic Stimulator (MagPro R30, Atlanta, GA) with an active/placebo figure-8 research coil (Cool-B65 A/P Coil) was used for all transcutaneous magnetic stimulation sessions. The spinous processes of the lower vertebrae in each subject were palpated, and thoracic 11 to lumbar 4 vertebrae were marked. The coil was centered along the midline at the L1 vertebral level during the stimulation and oriented such that the magnetic field generated was parallel to the spinal cord (rostral-caudal). We used trains of biphasic, single pulse (duration 250 µs), continuous, magnetic stimulation. Each stimulation session consisted of three 4-min continuous stimulation periods with a 30 second break between each stimulation period for a total of 13 minutes (a total of 12 minutes of stimulation plus 1 minute of breaks). For the first two weeks, each subject underwent stimulation at 1 Hz and 30 Hz frequencies (week one: 1 Hz/30 Hz/1 Hz, and week two: 30 Hz/1 Hz/30 Hz) until the better frequency was determined for the patient at the first follow-up UDS after the 2^nd^ week of stimulation. The frequency of 1 Hz and 30 Hz was selected based on our previous work in animals and humans^12,14^. Changes in urethral (directly measured) and detrusor (vesicular – abdominal) pressures during micturition attempts were measured during both low frequency stimulation (1 Hz) and high frequency stimulation (30 Hz). The stimulation frequency that resulted in the combination of increased detrusor pressure and decreased urethral pressure during attempted micturition (hence, promoting bladder emptying) was selected as optimal. The intensity of stimulation was set 20% below the intensity that elicited local paraspinal muscular contraction for each subject (since muscle contractions would have unmasked the double blinding). This stimulation intensity was usually around 40–50% of the maximal field strength of 2 Tesla. Once the optimal frequency was determined, all subjects received the optimal stimulation frequency only at a constant intensity for the remaining 16, weekly bladder rehabilitation sessions.

### Electrophysiology

At the end of the assessment phase, the following electrophysiological data were obtained on each subject before, during and after low frequency (1 Hz) and high frequency (30 Hz) transcutaneous magnetic stimulation: bulbocavernosus reflex (BCR), electromyography (EMG) and spinal evoked potentials (SEP) bilaterally in the pelvic floor and in the vastus lateralis, gastrocnemius, gluteus and quadriceps femoris muscles.

Pelvic floor EMGs were obtained using needle electrodes (Laborie 1512A-M, Laborie International, Mississauga, ON, Canada). All other muscle EMGs were obtained with 1 inch surface pad electrodes (MultiBioSensors, El Paso, TX).

The BCR was obtained by using ring stimulating electrodes (Cadwell 302243-200, Cadwell Industries, Kennewick, WA) that were stimulated with a monophasic electric pulse at 1.5 Hz, pulse width 0.2 ms, and intensity at three times the sensory threshold (or 35 mA if the subject had no sensation). At least 100 pulses were given in each BCR test session.

Recording, amplification and digitization of all data were done using an RZ2 amplifier and a PZ5–32 TDT digitizer (Tucker Davis Technologies, Alachua, FL) with a 60 Hz notch filter and band pass filtering to exclude frequencies <3 Hz and > 200 Hz.

### Data Analysis

The primary outcome was voluntary urination volume per day. Pre-specified secondary outcomes included urine stream flow rate, bladder capacity, catheterizations per day, sexual health inventory for men (SHIM), and urinary incontinence quality of life scale (iQOL). All electrophysiological data from the TDT system (Tucker Davis Technologies, Alachua, FL) were exported to a computer and analyzed using MatLab (Matlab2015b, MathWorks, Natick, MA). The BCR amplitudes and latency were calculated for every single electrical pudendal stimulation. Spinal evoked potentials (if present) were identified in the continuous recording of lower extremity EMGs.

Urodynamic data were exported from the Laborie system and analyzed using Microsoft Excel (Excel2010, Microsoft, Redmond, WA). The changes in urethral pressure (P urethra) and detrusor pressure (P detrusor) were measured and compared during baseline and during attempted micturition.

Statistical significance was assessed with Analysis of Variance (ANOVA) and paired Student’s T-tests and the Bonferroni correction for multiple preplanned comparisons, when appropriate, using R 3.25 (https://www.r-project.org) and Graphpad Prism (Graphpad Software, La Jolla, CA), respectively.

### Data Availability

All data generated during and/or analyzed during the current study are available from the corresponding author on reasonable request. For further details of study protocol please see Supplementary Information.

## Electronic supplementary material

Clinical Protocol
