# Supplementary material for: A Proof-of-Concept Study of Transcutaneous Magnetic Spinal Cord Stimulation for Neurogenic Bladder
Source: Sci Rep. 2018 Aug 22;8:12549. doi: 10.1038/s41598-018-30232-z (PMC6105631; doi:10.1038/s41598-018-30232-z)
Supplement: Supplementary file 1 — Clinical Protocol [file 41598_2018_30232_MOESM1_ESM.docx]

**Restoring Bladder Function by Spinal Cord Neuromodulation in SCI**

**Protocol # 001**

**IRB#14-000932**

**Approval Date: 4/13/2015**

**PI: Daniel C. Lu, MD. PhD**

Table of Contents

1. **Background and significance**3
2. **Objective**3
3. **Subject population**3
   1. Subject recruitment4
   2. Subject enrollment4

3.21 Inclusion criteria …………………………………………………………………………………………………………..4

3.21 Exclusion criteria ………………………………………………………………...………………………………………..4

1. **Study plan**4
   1. Phase of study4
   2. Sequence and timing5
   3. Protect privacy6
   4. Testing locations6
2. **Data analysis**7
   1. Statistical analysis7
   2. Subject confidentiality7
3. **Potential risks**7
   1. Risk from transcutaneous magnetic stimulation7
   2. Risk from interventions and experimental procedures8
4. **Potential benefits**8
   1. Subject benefit8
   2. Societal benefit8
5. **Investigator responsibilities**9
6. **Appendices**9
7. **References**9
8. **Background and Significance:**

Little progress has been made in developing any intervention that will enhance bladder function after a SCI. However our team has performed a systematic progression of experiments in animals and patients showing that several forms of electrical spinal cord stimulation can detect and improve spared function. We have implanted an epidural stimulation (EDS) electrode array over the lumbosacral spinal cord in 4 human subjects with a motor complete SCI. Each has gained some voluntary control of urinary voiding in the absence of EDS (1, 2). Furthermore, we have preliminary data showing that single-electrode TESS (transcutaneous electric spinal cord stimulation) and TMS (transcutaneous magnetic stimulation) of the cervical spinal cord can improve fine motor function of the upper limb in human subjects with incomplete quadriplegia. The critical question here is whether TESS or TMS can be used to enable spared function of sacral neuromotor networks, i.e., neural networks related to bladder function in animals and humans (3-6). The potential impact of these therapeutic interventions on the lives of individuals with urinary incontinence cannot be overestimated (7-9). Development of magnetic stimulation to activate spared, but silent, spinal cord pathways related to bladder function in humans could represent the beginning of a paradigm shift in the rehabilitative approach to bladder incontinence as a result of SCI and potentially other neurologic injuries or stroke (10-13). It could also provide new pathways toward more advanced technologies to further enable more potential success in improving bladder function.

As we have observed with studies of the lumbosacral spinal cord in completely paralyzed SCI subjects, future development of technical capabilities to use neuromodulation of the lumbosacral spinal cord for evaluation and enabling of spared function will undoubtedly enhance our ability to improve therapies for bladder function after paralysis. By discovering spared function and revealing the potential for treatments, the cost-savings from the proposed translational studies could include reduced assistive daily care costs, increased employment, and improved quality of life—especially for incomplete SCI patients who account for the majority of these SCI patients. The proposed studies could provide a partial solution to the US$40 billion/year care and $5.5 billion/year lost productivity costs (11, 14). Of the 10 million people in the US living with paralysis, 15,000 are the result of a SCI each year. The first year of care can range from $322,000-$986,000, with lifetime costs of $1.4-4M for someone injured at 25 years of age. In addition to potentially devastating sensorimotor disturbances, there is a huge financial cost of SCI, estimated to be $13.55B in medical care, therapy, and lost productivity nationwide. This proposal may positively impact patients with chronic or new SCI and other forms of nervous system damage or disease (stroke, multiple sclerosis).

1. ***Objectives***

The proposed study is to investigate the effectiveness of transcutaneous magnetic spinal cord stimulation can improve chronic spinal cord injured patients’ urinary function.

The pilot study is designed to enroll five subjects. It is designed with each subjects serving as their own control.

Hypothesis: transcutaneous magnetic stimulation can enable lumbosacral spinal circuitry to enable bladder control in chronic spinal cord injured individuals.

1. ***Subject Population***

3.1 Subject recruitment:

Recruitment of individuals with SCI will be from VA Greater Los Angeles, VA Long Beach, and UCLA Health Care System. Additional referrals will be generated from treating physiatrists, urologist, neurologists, and other clinicians at referral sites.

Initial contact will be made in person by a potential participant's physiatrist, urologist, neurologists or other clinician during an office visit. The potential participant will be provided with contact information, e.g. phone number, email address, of the PI, Dr. Lu. Once Dr. Lu has been contacted, he and his staff will schedule a visit by the potential subject for potential consent and enrollment.

3.2 Subject enrollment:

Prior to enrollment, the potential subject will be contacted by the PI. A phone questionnaire will be completed to ensure potential candidacy. The potential candidate will then be scheduled a visit to review the records and sign the informed consent in order to enroll.

3.21 Inclusion criteria

- Male 18-75 years; This is required to have one urethral anatomy. Secondarily, more males have spinal cord injury, especially in veteran populations.
- At least 1 year post-injury;
- Non-progressive SCI at C2-T8 (non-conus injury);
- Motor Complete ISNCSCI ASIA Impairment Scale;
- Neurogenic bladder requiring clean intermittent straight catheterization;
- Able to attend weekly testing sessions for 6 months.
- Have intact lower extremity anatomy and able to use lower extremity for assistive standing and stepping. This is required to assess the quality of motor-function-activating spinal cord stimulation.

3.22 Exclusion criteria

- History of autonomic dysreflexia;
- Ventilator dependency;
- Musculoskeletal dysfunction, unhealed fracture, pressure ulcer, active infection;
- Clinically significant depression or ongoing drug abuse;
- Received botox injection, or bladder surgery (suprapubic access, Brindley procedure, etc.); prostatic hypertrophy or bladder outlet disorder;
- Cardiopulmonary disease that precludes lower extremity training or rehabilitation.

1. ***Study plan***
   1. *A description of each phase of the study. Details in Section 4.2.*

Phase # 1: Assessment—Define stimulation parameters that improve bladder function

Phase # 2: Treatment—Weekly stimulation to improve bladder function

Phase # 3: Follow-up—Determine the decay of stimulation effect

- 1. *Sequence and timing of study procedures to be performed.*

Phase # 1:

- - - - Urodynamic testing: Beginning and end
      - Bladder diary: Daily
      - Self assessment: SHIM, I-QOL: Beginning
      - Transcutaneous magnetic stimulation: weekly, 1Hz (low frequency) then 30Hz (high frequency)
      - ASIA Score: Beginning
      - Electrodiagnostic BCR: Beginning

Phase # 2:

- - - - Urodynamic testing: Start, monthly until conclusion (16 weeks)
      - Bladder diary: Daily
      - Self assessment: SHIM, I-QOL: end of phase
      - Transcutaneous magnetic stimulation: weekly with optimal stimulating parameter from Phase # 1
      - ASIA Score: End of the phase
      - Electrodiagnostic BCR: End of the phase

Phase # 3:

- - - - Urodynamic testing: Start (same as end of phase # 2)
      - Bladder diary: Daily
      - Self assessment: None
      - Transcutaneous magnetic stimulation: weekly sham
      - ASIA Score: End of phase
      - Electrodiagnostic BCR: none

**• Urodynamic testing** (a standard procedure for research purposes to determine bladder function):

Subject will be placed in supine position. Bladder will be emptied of existing urine by inserting a Foley catheter using aseptic technique and sterile equipment. Pre-warmed 37°C saline will be instilled at a rate of <30 mL/Min, to avoid any viscerosymapthetic reflex, until subject reports an urge to void, or 400 mL, whichever happens first. To avoid autonomic dysreflexia brought on by bladder filling (viscerosympathetic reflex), arterial blood pressure will be monitored and fluid infusion will be stopped after observing >10 mmHg increase in systolic and/or diastolic blood pressure and/or flushing. Intermittent ultrasound will be applied to assess any reflux and measure bladder distention. Rapid bladder emptying with Foley catheter will be performed if any of these changes was observed; if the symptoms do not improve nifedipine and nitrates (e.g., nitroglycerine paste or sublingual nitroglycerine) will be the next step of management. If there are any poor responses to treatment, the patient will be transferred to Ronald Reagan hospital for further management. Fluoroscopic imaging will be performed to avoid any vescicourethral reflux during the filling phase. Subject will then be asked to volitionally void without the use of any intervention for 5 minutes. Afterwards, the subject will be asked to void with the presence of transcutaneous or magnetic stimulation applied to the skin overlying the subject’s lower thoracic or lumbosacral spine for 5 minutes. Voided volume will be collected in a 1000 mL graduated plastic urinal. For the duration of the urodynamics testing, fluoroscopic imaging of the bladder and urethral sphincters will be performed intermittently, and rectal pressure, intravesicular pressure, external urethral sphincter and detrusor muscle pressures will be monitored and recorded. Upon completion of the test, the remaining saline instilled in the bladder will be emptied and recorded as residual volume. Duration: 60 minutes

**• Transcutaneous Magnetic Stimulation** (experimental procedure to attempt to activate *sacral parasympathetic nucleus (SPN) that contain parasympathetic preganglionic neurons* bladder-related circuits in the spinal cord): application stimulation coil to skin over the lower thoracic or lumbosacral cord spinal column (centered on midline lumbar-1 level). Application of 1-*30* Hz of stimulation. Stimulation amplitude will start at 1% Power (Magnetic stimulation) and will be increased in increments 1% Power (Magnetic stimulation) until the subject reports an urge to void or max tolerance. Maximum amplitude will not exceed 100% Power (Magnetic stimulation). Stimulation will be administered for 4 minute intervals at 3 times per session with 30 second break between intervals. Duration: 15-20 minutes

**• Self-Assessment** (standard procedures for research purposes): Incontinence Quality of Life (I-QOL), Sexual Health Inventory for Men (SHIM). Duration: 15 minutes

**• Bladder Diary** (standard procedure for research purposes): Subject will be asked to record voiding by catheterization or without catheterization. Number of accidents and bladder infections will be recorded, as well. Bladder diary will be performed at home. Duration 1-2 minutes

- 1. *How we will protect the privacy of the subjects during data collected.*

Patient will be consented in the clinic in a private room, behind closed doors. Subsequent testing will be conducted in a clinical research laboratory space in UCLA CTRC. All data collected will be placed on password protected files on an encoded hard drive. The data will be de-identified and coded key placed in a password protected file and on a separate encoded hard drive. The hard drives will be placed in a locked cabinet in the office of the PI in CHS, the door to the office is locked with only access from the PI, CHS is located behind security access points during off hours. Personally identifying information will be coded. Coding will be performed by a random number generator and code assigned to study participants. The coded key will be kept on a printout in a locked file cabinet behind a locked office door in CHS.

*• The approximate duration, intervals of administration and overall length of participation.*

1 hour for recruitment, 3 hours for enrollment. The study will take place over 24 weeks with weekly stimulation/testing and each session will take about 20 minutes – 1hr. 100 hours total.

- 1. *Testing locations:*

UCLA Clinical Translational Research Center (CTRC)

UCLA Medical Plaza 200, 6^th^ Fl, Ambulatory Surgery Center

1. ***Data Analysis***

*5.1 Statistical analysis:*

All electrophysiological data from the TDT system (Tucker Davis Technologies, Alachua, FL) were exported to a personal computer and analyzed in MatLab (Matlab2015b, MathWorks, Natick, MA). The BCR amplitudes and latency were calculated for every single electrical pudendal stimulation. Spinal evoked potentials (if present) were identified in the continuous recording of lower extremity EMGs.

Urodynamic data were exported from the Laborie system to a personal computer and analyzed in Microsoft Excel (Excel2010, Microsoft, Redmond, WA). The changes in urethral pressure (P urethra) and detrussor pressure (P detrussor) were measured and compared during baseline and during attempted micturition.

Statistical significance was assessed with Analysis of Variance (ANOVA) and paired Student’s T-test using R 3.25 (https://www.r-project.org) and Graphpad Prism (Graphpad Software, La Jolla, CA), respectively.

5.2 Subject confidentiality

Subject confidentiality will be maintain throughout the study. All clinical and research data gathered will be de-identified and stored in a secured location. Digital data will be stored on a secured computer that can only be accessed by the authorized individuals. The data will be backed up onto an encrypted and secured hard drive.

In addition, Dr. Daniel Denis and Dr. Niu will be responsible for overseeing the study safety, along with the External Research Monitor, Victor Chang, MD. The Research Monitor, Victor Chang, MD (Director, Spine Research, Department of Neurosurgery, Henry Ford West Bloomfield Hospital, West Bloomfield, Michigan) is responsible to oversee the safety of the research and report observations/findings to the IRB of Record or a designated official. The Research Monitor will review all unanticipated problems involving risk to volunteers or others associated with the protocol and provide an unbiased written report of the event to the IRB of Record. The Research Monitor may discuss the research protocol with the investigators, interview human subjects, and consult with others outside of the study about the research. The Research Monitor shall have authority to stop the research protocol in progress, remove individual human subjects from the study, and take whatever steps are necessary to protect the safety and well-being of human subjects until the IRB can assess the monitor's report. The Research Monitor is responsible for promptly reporting their observations and findings to the IRB.

1. ***Potential risks***

6.1 Risk from Transcutaneous Magnetic Stimulation: The transcutaneous stimulation device is noninvasive and procedure has been approved by the UCLA Institutional Review Board (IRB#11-001720). There is a minor risk of discomfort during the stimulation procedure that stops after stimulation. There is also a minor risk of skin irritation with the adhesive electrode during stimulation/recording. If this occurs, another site can be used, or testing postponed until skin heals.

6.2 Risk from Interventions and Experimental Procedures: Because subjects must meet the criteria listed above, we expect all subjects to be in good health. The studies described may involve the following physical risks and/or discomforts: 1) increased respiration or shortness of breath; 2) increased heart rate; 3) muscle and joint soreness; 4) lowering or elevation of blood pressure; 5) dizziness; 6) skin irritation from recording electrodes, or hand placements of trainers; 7) skin abrasion from hand placements of trainers; and 8) muscle strain or joint sprain from movement, or from the force exerted by the trainers.

It is highly unlikely that a subject would feel chest pain or high blood pressure would occur that did not resolve within several minutes. These events have not occurred in our past experience. Blood pressure will be monitored throughout the testing session at 1-5 minute intervals by arm blood pressure cuff. However, if this did occur the individual would be immediately transported to the University of California, Los Angeles Emergency Unit and Drs. Lu, and/or Denis, and/or Niu notified.

There may be a risk of autonomic dysreflexia that may cause hypertension and headaches due to increase in bladder volume during urodynamic testing. This usually resolves after immediate evacuation of bladder fluid. If blood pressure is persistently high, topical nitroglycerine or nifedipine may be administered. The CTRC is equipped with pharmacy, nursing, and code staff to provide immediate support. The likelihood of this occurring is very low if patient has no previous history of autonomic dysreflexia, which is an exclusionary criterion for this study.

There is a risk of urodynamic study during testing due to fluoroscopy use. There is a small risk of development of cancer from x-ray used during the monthly formal video urodynamic testing, however this risk is small. The aggregate dose of exposure is approximately 1 chest x-ray during each session. This dose is low and below limits and is not expected to be harmful.

There is a risk of urinary tract infection due to the catheterization during the urodynamic procedure. However all efforts are used to minimize this risk, such as use of sterile technique. Additionally, this risk of urinary tract infection with the urodynamic testing should not exceed the infection risk of daily catheterization and bladder care.

There are no identifiable psychological, sociological, economical, or legal risks to the subjects.

1. ***Potential benefit***

7.1 Subject benefit: our hypothesis is that subjects will be able to have improved bladder control with transcutaneous magnetic stimulation. The subjects will therefore require fewer catherization and hence, lower likelihood of infection or traumatic injuries to the urinary tract. Furthermore, the subjects may experience improves sexual functions and bowel control.

7.2 Societal benefit: Importance of the Knowledge to Be Gained. The proposed experiments will demonstrate whether a new strategy of neuromodulation via non-invasive spinal cord stimulation can be used to improve bladder function. Positive demonstration of the proof-of-principle of the neuromodulatory strategy would almost certainly result in significant improvements in the quality of life after a spinal cord injury and could significantly reduce the cost of healthcare for these individuals by making them more independent. The knowledge gained also will demonstrate whether a medical stimulation device that is presently approved for other neuromotor dysfunctions can be used to improve bladder function. In addition we will learn whether a newly developed technology, transcutaneous stimulation, can be used to neuromodulate the spinal cord to improve bladder function. To date, there has been virtually no progress in improving bladder function after spinal cord injuries. The potential of the proposed studies are extremely positive. Given the magnitude of the scientific evidence from which the neuromodulatory strategy has evolved combined with our preliminary evidence in humans and rats, the potential gain that can be realized by so many impaired individuals given the modest total cost to be incurred in this grant cannot be denied.

1. ***Investigator responsibilities***

The principle investigator will be required to sign investigators agreement and adhere to the following:

- The protected health information requested is the minimum necessary to meet the research objectives
- The protected health information that is obtained as part of this study will not be used or disclosed to any other person other than study personnel except as required by law.
- Study Sponsors will not be provided with personal identifying information (including PHI) to take from the study site at any time, including the end of the study.
- Data and specimens shared with outside entities, such as study sponsors, will be coded or de-identified.

1. ***Appendices***

Appendix A: Telephone screening

Appendix B: Informed consent

Appendix C: Incontinence-Quality of Life (I-QOL) questionnaire English version

Appendix D: Sexual Health Inventory for Men (SHIM) questionnaire English version

Appendix E: American Spinal Injury Association Score English version

Appendix F: Standard Bladder Diary

1. ***References***

1. Harkema S, Gerasimenko Y, Hodes J, Burdick J, Angeli C, Chen Y, et al. Effect of epidural stimulation of the lumbosacral spinal cord on voluntary movement, standing, and assisted stepping after motor complete paraplegia: a case study. Lancet. 2011;377(9781):1938-47. Epub 2011/05/24. doi: 10.1016/S0140- 6736(11)60547-3. PubMed PMID: 21601270; PubMed Central PMCID: PMC3154251.

2. Angeli. Improvements in level of recovery of task specific voluntary control of lower limbs with lumbosacral epidural stimulation and training after chronic complete paralysis. 2012 Neuroscience Meeting Planner Washington, DC: Society for Neuroscience, 2012 Online

3. Alaynick WA, Jessell TM, Pfaff SL. SnapShot: spinal cord development. Cell. 2011;146(1):178- e1. Epub 2011/07/07. doi: 10.1016/j.cell.2011.06.038. PubMed PMID: 21729788; PubMed Central PMCID: PMC3158655.

4. Gallarda BW, Sharpee TO, Pfaff SL, Alaynick WA. Defining rhythmic locomotor burst patterns using a continuous wavelet transform. Ann N Y Acad Sci. 2010;1198:133-9. Epub 2010/06/12. doi: 10.1111/j.1749- 6632.2010.05437.x. PubMed PMID: 20536927.

5. Garudadri S, Gallarda B, Pfaff S, Alaynick W. Spinal cord electrophysiology II: extracellular suction electrode fabrication. J Vis Exp. 2011(48). Epub 2011/03/05. doi: 10.3791/2580. PubMed PMID: 21372792.

6. Meyer A, Gallarda BW, Pfaff S, Alaynick W. Spinal cord electrophysiology. J Vis Exp. 2010(35). Epub 2010/01/20. doi: 10.3791/1660. PubMed PMID: 20084054; PubMed Central PMCID: PMC2841572.

7. Anderson KD. Targeting recovery: priorities of the spinal cord-injured population. J Neurotrauma. 2004;21(10):1371-83. Epub 2005/01/28. PubMed PMID: 15672628.

8. Alexander MS, Anderson KD, Biering-Sorensen F, Blight AR, Brannon R, Bryce TN, et al. Outcome measures in spinal cord injury: recent assessments and recommendations for future directions. Spinal Cord. 2009;47(8):582-91. Epub 2009/04/22. doi: 10.1038/sc.2009.18. PubMed PMID: 19381157; PubMed Central PMCID: PMC2722687.

9. National Spinal Cord Injury Statistical Center. Spinal cord injury facts and figures at a glance. J Spinal Cord Med. 2014 Jul;37(4):479-80. doi: 10.1179/1079026814Z.000000000322.

10. Edgerton VR, Roy RR. A new age for rehabilitation. Eur J Phys Rehabil Med. 2012. Epub 2012/03/13. PubMed PMID: 22407010.

11. Edgerton VR, Harkema S. Epidural stimulation of the spinal cord in spinal cord injury: current status and future challenges. Expert Rev Neurother. 2011;11(10):1351-3. Epub 2011/10/01. doi: 10.1586/ern.11.129. PubMed PMID: 21955190.

12. Fong AJ, Roy RR, Ichiyama RM, Lavrov I, Courtine G, Gerasimenko Y, et al. Recovery of control of posture and locomotion after a spinal cord injury: solutions staring us in the face. Prog Brain Res. 2009;175:393-418. Epub 2009/08/08. doi: 10.1016/S0079-6123(09)17526-X. PubMed PMID: 19660669; PubMed Central PMCID: PMC2904312.

13. Gerasimenko Y, Roy RR, Edgerton VR. Epidural stimulation: comparison of the spinal circuits that generate and control locomotion in rats, cats and humans. Exp Neurol. 2008;209(2):417-25. Epub 2007/09/14. doi: 10.1016/j.expneurol.2007.07.015. PubMed PMID: 17850791; PubMed Central PMCID: PMC2288525.

14. Bailey J, Dijkers MP, Gassaway J, Thomas J, Lingefelt P, Kreider SE, et al. Relationship of nursing education and care management inpatient rehabilitation interventions and patient characteristics to outcomes following spinal cord injury: the SCIRehab project. J Spinal Cord Med. 2012;35(6):593-610. Epub 2013/01/16. doi: 10.1179/2045772312Y.0000000067. PubMed PMID: 23318039; PubMed Central PMCID: PMC3522899.
